# Evidence for Functional Diversity between the Voltage-Gated Proton Channel Hv1 and Its Closest Related Protein HVRP1

**DOI:** 10.1371/journal.pone.0105926

**Published:** 2014-08-28

**Authors:** Iris H. Kim, Peter Hevezi, Csaba Varga, Medha M. Pathak, Liang Hong, Dennis Ta, Chau T. Tran, Albert Zlotnik, Ivan Soltesz, Francesco Tombola

**Affiliations:** 1 Department of Physiology and Biophysics, University of California Irvine, Irvine, California, United States of America; 2 Department of Anatomy and Neurobiology, University of California Irvine, Irvine, California, United States of America; University of Hull, United Kingdom

## Abstract

The Hv1 channel and voltage-sensitive phosphatases share with voltage-gated sodium, potassium, and calcium channels the ability to detect changes in membrane potential through voltage-sensing domains (VSDs). However, they lack the pore domain typical of these other channels. Na_V_, K_V_, and Ca_V_ proteins can be found in neurons and muscles, where they play important roles in electrical excitability. In contrast, VSD-containing proteins lacking a pore domain are found in non-excitable cells and are not involved in neuronal signaling. Here, we report the identification of HVRP1, a protein related to the Hv1 channel (from which the name Hv1 Related Protein 1 is derived), which we find to be expressed primarily in the central nervous system, and particularly in the cerebellum. Within the cerebellar tissue, HVRP1 is specifically expressed in granule neurons, as determined by in situ hybridization and immunohistochemistry. Analysis of subcellular distribution via electron microscopy and immunogold labeling reveals that the protein localizes on the post-synaptic side of contacts between glutamatergic mossy fibers and the granule cells. We also find that, despite the similarities in amino acid sequence and structural organization between Hv1 and HVRP1, the two proteins have distinct functional properties. The high conservation of HVRP1 in vertebrates and its cellular and subcellular localizations suggest an important function in the nervous system.

## Introduction

**Figure 1:**
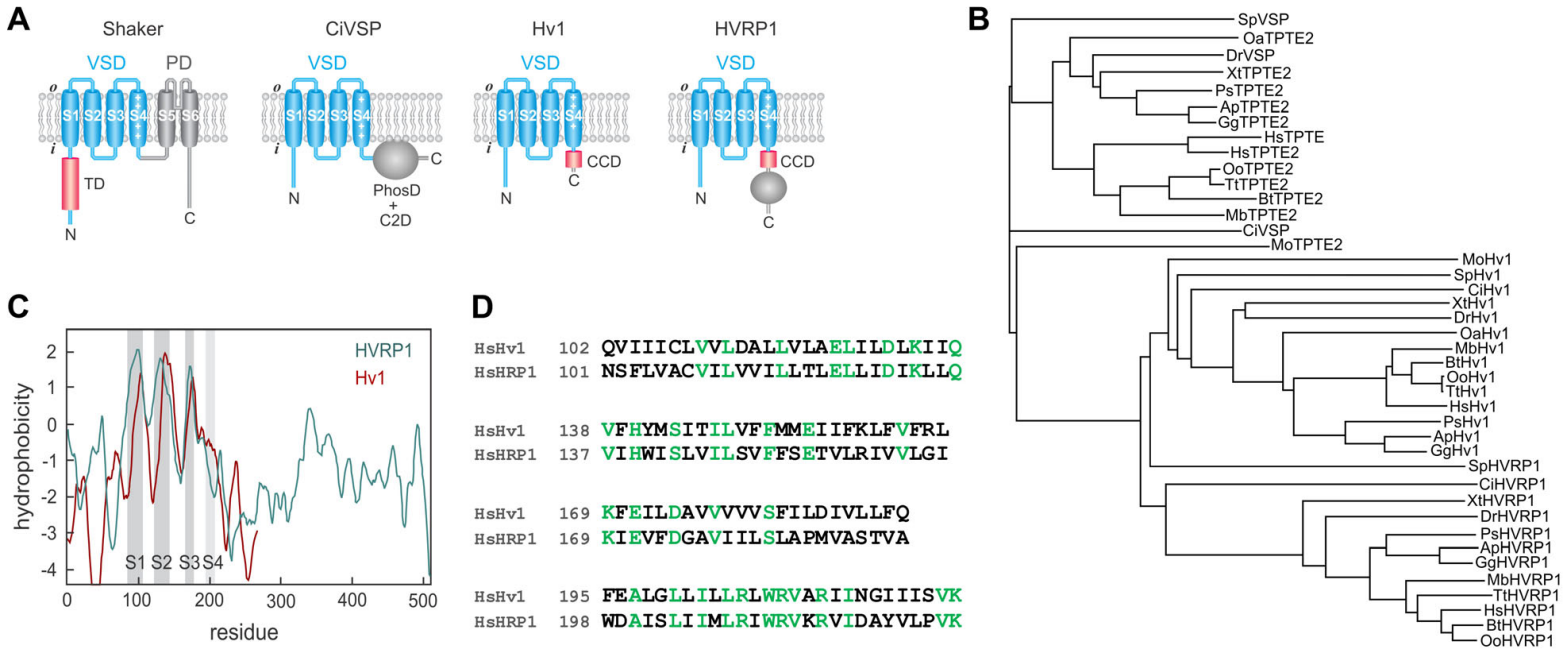
Relationship between HVRP1 and other VSD-containing proteins. **A**) Modular organization of HVRP1 compared to known voltage sensing proteins. The Shaker potassium channel was chosen as an example of protein containing both a VSD and a pore domain. C2D: C2 domain, CCD: coiled-coil domain, PhosD: phosphatase domain, PD: pore domain, TD: tetramerization domain. **B**) Phylogram showing amino acid sequence relationship between full-length VSP/TPTE/TPTE2 phosphatases, Hv1 channels, and HVRP1 proteins. See Methods for details. Ap: *Anas platyrhynchos*, Bt: *Bos taurus*, Ci: *Ciona intestinalis*, Dr: *Danio rerio*, Gg: *Gallus gallus*, Hs: *Homo sapiens*, Mb: *Myotis brandtii*, Mo: *Metaseiulus occidentalis*, Oa: *Ornithorhynchus anatinus*, Oo: *Orcinus orca*, Ps: *Pelodiscus sinensis*, Sp: *Strongylocentrotus purpuratus*, Tt: *Tursiops truncates*, Xt: *Xenopus tropicalis*. **C**) Hydrophobicity plot (generated with TopPred, Institute Pasteur, [53]), comparing human Hv1 and HVRP1 and showing relative positions of transmembrane helices S1–S4 (identified with SOSUI, Nagoya University, [54]). **D**) Sequence alignment of human Hv1 and HVRP1. Only regions containing segments S1 through S4 are shown.

The molecular devices responsible for the generation and propagation of electrical signals in excitable tissues are proteins containing voltage-sensing domains (VSDs) [1]. Malfunction of such proteins is the cause of several neurological and cardiovascular diseases, such as epilepsy, episodic ataxia, migraine, periodic paralysis, and cardiac arrhythmia [2]. VSDs are four-transmembrane-segment structural units whose function is to turn on and off biological processes in response to changes in cell membrane potential. In most VSD-containing proteins, such as voltage-gated sodium, potassium, and calcium channels, the voltage sensor controls the opening and closing of an ion-conducting pore domain (Fig. 1A). Only recently have VSDs been recognized to perform other functions besides controlling pore domains. Two classes of proteins have been identified so far, which contain VSDs but do not possess pore domains: voltage-sensitive phosphatases (VSPs) [3] and voltage-gated proton (Hvs) channels [4], [5].

In the first class, the VSD is connected to a cytoplasmic enzymatic domain that turns on when the membrane is depolarized and dephosphorylates phosphatidylinositol (PI) lipids [3], [6] (Fig. 1A). CiVSP from *Ciona intestinalis* has been characterized in detail [7] and proven to be a useful tool to change PI-4,5-bisphosphate concentration in cells in a voltage dependent manner, e.g. [8], [9]. Its human homologs (TPTE and TPTE2/TPIP [10], [11], [12]) have been reported to be associated with intracellular compartments [10], [11]. Based on their catalytic activity and tissue distribution, voltage-sensitive phosphatases have been proposed to play a role in linking changes in membrane potential to phosphoinositide signaling pathways in non-excitable cells [13], [14], [15].

In the second class, represented by the voltage-gated proton channel Hv1 [4], [5] (a.k.a. HVCN1 or VSOP), the VSD acts both as a sensor of membrane potential and as an ion conducting unit, allowing protons to permeate the membrane upon depolarization (Fig. 1A). Hv1 forms dimers in which each VSD subunit has its own proton pore and gate [16], [17], [18]. Its cytoplasmic C-terminal coiled-coil domain [19], [20] is responsible for dimerization, and the two Hv1 subunits open cooperatively [21], [22], [23]. Hv1 is known to play important roles in various non-excitable tissues, where it counteracts intracellular proton accumulation and regulates the production or reactive oxygen species (ROS) by NOX enzymes [24]. In the brain, the channel is expressed in the microglia [25], [26], and its excessive activity has been shown to worsen recovery from ischemic stroke [25].

Proton currents from molecularly unidentified voltage-gated channels were first recorded in snail neurons more than thirty years ago [27]. These currents resemble those produced by vertebrate Hv1 channels. However, there is strong evidence that Hv1 is not a neuronal protein in vertebrates [25], [26], [28]. We wondered whether VSD-containing proteins similar to Hv1 could exist in vertebrate neurons and play a role in cell electrical excitability.

Using a combination of bioinformatics, gene expression profiling, *in situ* hybridization, immunohistochemistry, electron microscopy, and electrophysiology we identified the product of the gene *C15ORF27* as a neuronal VSD-containing protein lacking the pore domain related to the Hv1 channel. We refer to this protein as HVRP1 (*HV*1 *R*elated *P*rotein 1). Based on the cellular and subcellular localization of the protein, and the functional comparison between Hv1, HVRP1, and chimeras made of parts of the two proteins, we propose that HVRP1 plays a role in the modulation of postsynaptic excitability in cerebellar granule neurons, and that despite its sequence similarity to the Hv1 channel, its function does not involve VSD-mediated ion permeation.

## Results

To identify proteins similar to Hv1, we performed a search with BLASTP [29], using the sequence of the VSD and C-terminus of the human Hv1 protein as query. We then reduced the number of hits by imposing that the protein: 1) should be made of four transmembrane segments, 2) should possess a coiled-coil domain in the cytoplasmic region, and 3) should not be yet characterized. The predicted protein from the human gene *C15ORF27* was found to be a promising candidate (identity: 26%, coverage: 99%) in agreement with an earlier report in which the sequence similarity between HVRP1 and Hv1 was determined using a distinct approach [30].

The hydrophobicity plot of C15orf27/HVRP1 indicates that the protein lacks the S5 and S6 transmembrane segments typical of Na_V_, K_V_, and Ca_V_ channels (Fig. 1C). In Hv1, the cytoplasmic C-terminus is rather short (∼50 a. a.) and it is composed solely of a coiled-coil domain starting right after the fourth transmembrane segment. In HVRP1, the C-terminus is as large as in CiVSP (∼300 a. a.), and it contains a predicted coiled-coil domain similar in length and position to the coiled-coiled domain of Hv1 (Fig. S1).

Overall, the HVRP1 protein is more tightly related to Hv1 than to voltage-sensitive phosphatases (Fig. 1B) and it is highly conserved in vertebrates (e.g., human and zebrafish HVRP1 sequences are 60% identical). The evolutionary relationship between different VSD-containing proteins has been recently investigated [30], [31], [32], and the VSDs of Hv1 and C15orf27/HVRP1 were found to be more closely related to Na_V_ channels than to other voltage-gated ion channels. The arginine-repeat motif in S4, responsible for voltage sensing, is highly conserved between Hv1 and HVRP1, as are negatively charged residues in S2 and S3 known to form salt bridges with the S4 arginines [33] (Fig. 1D). The Hv1 aspartate residue in S1 involved in the cooperative gating of the channel’s two subunits [34] is conserved in HVRP1, and so is the S2 histidine that contributes to binding zinc in Hv1 [4], [35] (Fig. 1D). The VSDs of voltage-gated ion channels and voltage-sensitive phosphatases contain a signature phenylalanine in the middle of S2 known to play a key role in voltage sensing as charge transfer center [36], [37]. The phenylalanine appears to be highly conserved also in HVRP1 proteins (Fig. 1D).

### HVRP1 tissue distribution

**Figure 2:**
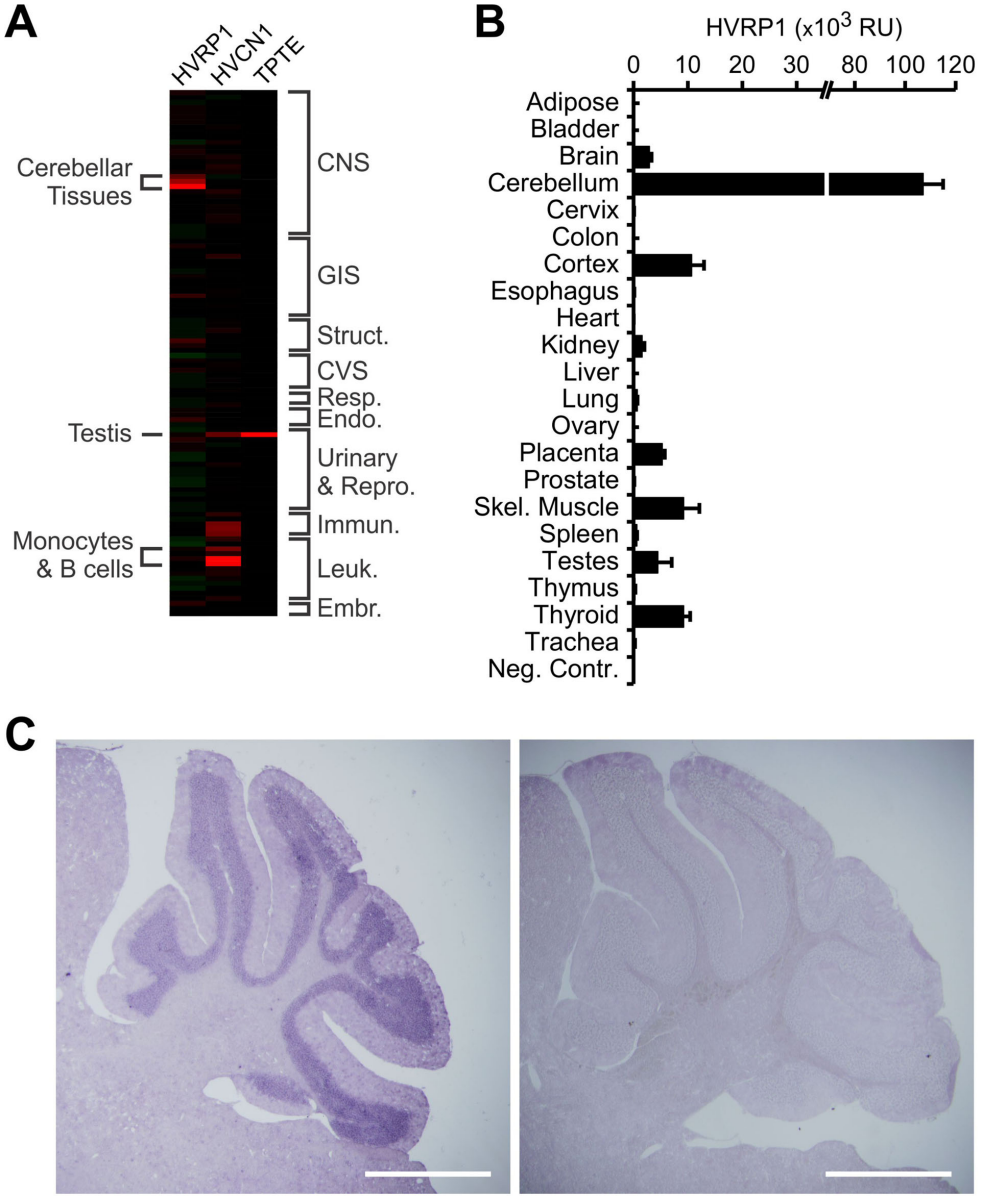
Tissue distribution of HVRP1 transcript. **A**) HVRP1 expression in human tissues assessed by Affymetrix microarray analysis. HVRP1 tissue distribution was compared to the distributions of human Hv1 (HVCN1) and TPTE. Red and green signals indicate up-regulated and down-regulated expression, respectively. Struct.: skeletal muscle, adipose tissue, skin. CVS: heart and blood vessels. Resp.: respiratory system. Endo.: endocrine organs. Urinary & Repro.: urinary & reproductive systems (male and female). Immun.: immune tissues. Leuk.: peripheral white blood cells. Embr.: embryonic tissues (see also Table S1). **B**) Levels of HVRP1 transcript in total RNA extracts from the indicated human tissues measured by qRT-PCR. Levels are reported as relative expression units (RU) in relation to the housekeeping control gene beta-actin. Water was used as negative control. Error bars are S.E.M., n = 3. **C**) *In situ* hybridization on a 20-µm thick sagittal section of an adult mouse brain cerebellar region, using a 600-bp riboprobe targeting the HVRP1 mRNA. Positively stained regions are dark purple. Left panel: antisense probe. Right panel: control sense probe. Scale bars: 1 mm.

The sequence similarity and the presence of a coiled-coil domain in both Hv1 and HVRP1 suggested that the two proteins could have similar physiological functions, or that they could work together as heteromultimers. A correlation in the tissue distribution between the two proteins would support this idea. So, we compared the tissue distribution of Hv1 and HVRP1 on a human genome-wide expression database produced using an Affymetrix genearray system that contained 54,675 probe sets per array, representing 21,974 unique Unigene clusters (i.e., more than 47,000 transcripts) [38], [39]. This body index of gene expression (BIGE) database compares gene expression across 105 normal tissues representing all major systems of the human body. In agreement with previous reports [4], [25], we found the highest levels of Hv1 transcript in the immune system and in the testis (Fig. 2A), and low expression in brain tissues. In contrast, we found HVRP1 primarily expressed in cerebellar tissues (Fig. 2A, Table S1). We also examined the expression profile of the TPTE (human homolog of CiVSP) and found it very different from the expression profile of HVRP1. We confirmed that the HVRP1 transcript is mainly present in the cerebellum by qRT-PCR on total RNA extracts from human tissues (Fig. 2B). HVRP1 expression was detected also in cerebral cortex, skeletal muscle, and thyroid, but at much lower levels.

### HVRP1 cellular and subcellular localization

**Figure 3:**
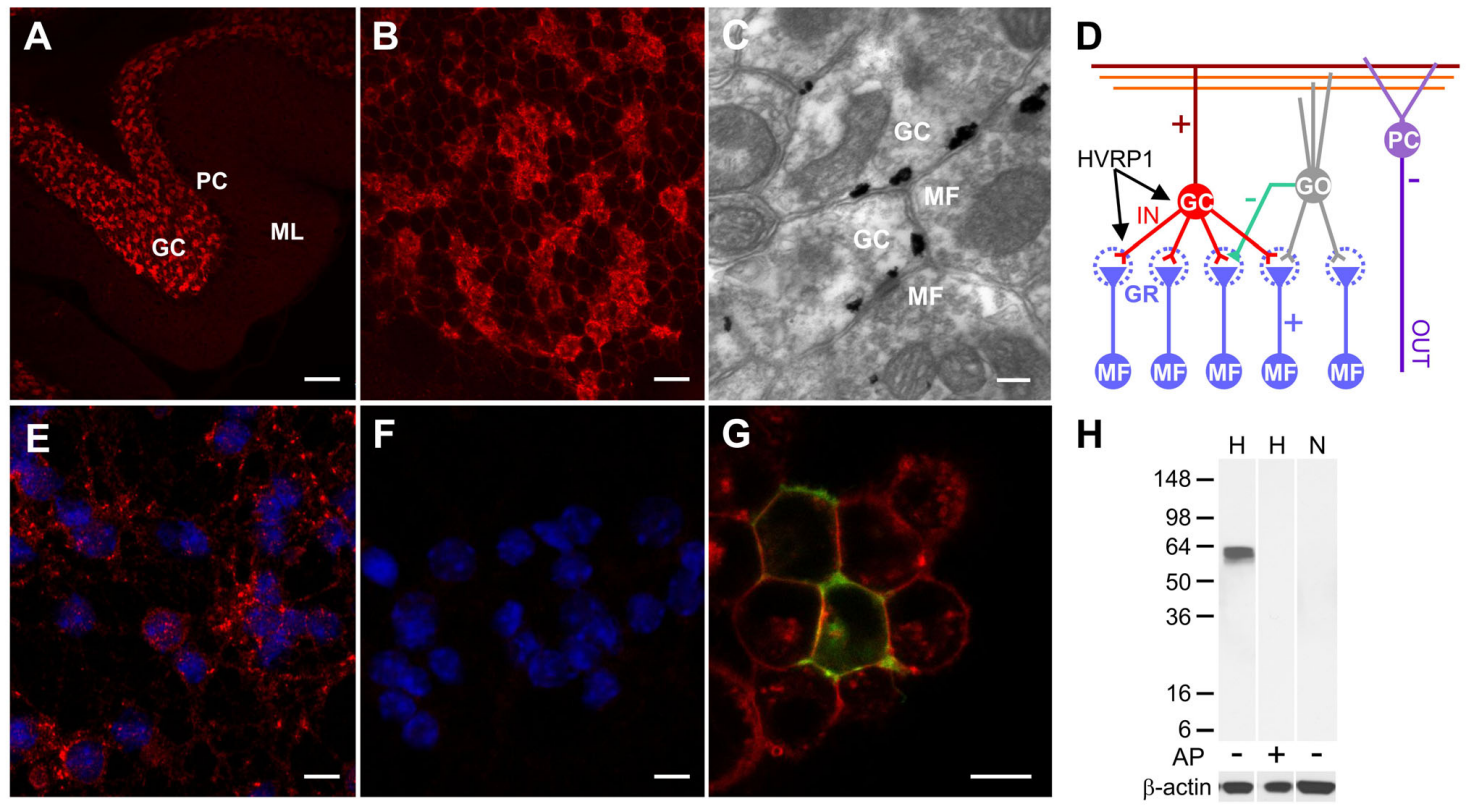
Cellular and subcellular localization of the HVRP1 protein. **A**) Immunohistochemical analysis of HVRP1 distribution in cerebellar tissue. Cerebellar cortical section from a wild-type rat stained with polyclonal antibody raised against HVRP1 (red). GC: granule cell layer, PC: Purkinje cell layer, ML: molecular layer, scale bar: 50 µm. **B**) Higher magnification of granule neurons expressing HVRP1, from (A). Scale bar is 10 µm. **C**) Ultrastructural localization of HVRP1 in the rat cerebellar GC layer visualized with the pre-embedding immunogold method. Positive labeling is in black, in the glomeruli, at the dendritic claws of granule cells surrounding mossy fiber terminals. Scale bar is 0.1 µm. **D**) Basic circuit diagram of the cerebellar cortex showing the location of HVRP1 in the dendrites and cell bodies of GCs. GO: Golgi cell, GR: glomerulus, MF: mossy fiber, PC: Purkinje cell. **E**) Confocal images of cultured mouse cerebellar granule neurons fixed and immunostained at 8 DIV. HVRP1 signal is shown in red (antibody dilution 1∶1000), DAPI signal is in blue. **F**) Staining as in (E) but with anti-HVRP1 antibody at dilution 1∶500 pre-incubated with HVRP1 antigen peptide (see Methods). Scale bars are 10 µm. **G**) Confocal image of live HEK293A cells expressing recombinant EGFP-tagged human HVRP1 (green) and labeled with the plasma membrane marker FM-464 (red). Scale bar is 10 µm. **H**) Western blot of total protein extracts from HEK293A cells transfected with hHVRP1 (H) and non transfected (N). AP indicates pre-incubation of anti-HVRP1 antibody with antigen peptide.

The cerebellum is made of well-characterized types of neurons and glial cells. To identify cerebellar cells expressing HVRP1, we performed *in situ* hybridizations on fixed cryosections of adult mouse brains. We detected high levels of HVRP1 mRNA in the cerebellar granule layer (Fig. 2C). We then investigated the cellular and subcellular localization of the HVRP1 protein by immunohistochemistry (IHC) and immunogold electron microscopy (EM) on fixed cryosections of adult rat brain, using a custom-made antibody raised against a C-terminal peptide of the mouse protein, which also recognizes human and rat HVRP1 (Fig. 3A–C). We found that the protein is present in the dendrites and soma of cerebellar granule neurons, but not in their axon (parallel fibers were not stained, Fig. 3). The immunogold EM analysis revealed the presence of HVRP1 within glomerular structures, on the postsynaptic side of the contacts between glutamatergic mossy fibers and granule cells (Fig. 3C–D).

The immunostaining of native HVRP1 indicated that the protein resides on the plasma membrane. We confirmed this finding by expressing recombinant HVRP1 in HEK293A cells and establishing its co-localization with the plasma membrane marker FM-4-64 (Fig. 3G). We detected the presence of the HVRP1 protein also in cultured granule neurons from P5–P6 neonatal mice assayed by immunocytochemistry (Fig. 3E–F). Western blots of HEK293A cells expressing human HVRP1 showed a band a 64 KDa (Fig. 3H). Preincubation of the primary antibody with the peptide antigen prevented protein recognition.

### Differences in functional properties between Hv1 and HVRP1

**Figure 4:**
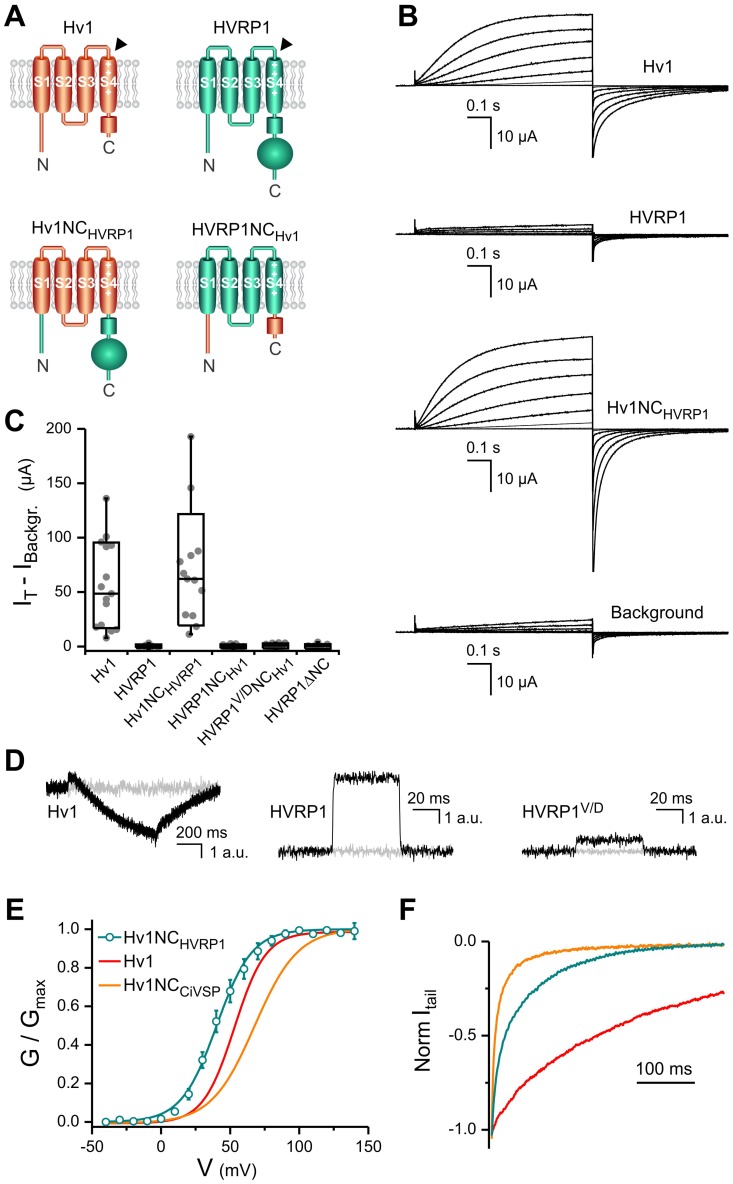
Comparison between the VSD conducting properties of Hv1 and HVRP1. **A**) Schematics of constructs used for the comparison (B–C). Black arrowheads indicate positions of the cysteine substitutions for fluorophore attachment (D). **B**) Proton currents from the indicated proteins expressed in a *Xenopus* oocyte measured in response to membrane depolarization by two-electrode voltage clamp. The test voltage was increased from 0 to +140 mV in 20 mV steps. Holding potential was −80 mV. Currents measured with HVRP1 are indistinguishable from the background. **C**) Quantification of test currents measured at +120 mV from traces like those shown in (B). Box indicates median ± S.D., whisker shows range. Individual measurements are shown as gray circles, n = 9–15. **D**) Fluorescence changes (expressed in arbitrary units) from TAMRA-MTS-labeled oocytes expressing the indicated proteins with or without cysteine substitution (shown in black and light gray, respectively). Substituted positions were: H193 in Hv1 and S196 in HVRP1. HVRP1^V/D^ contained the additional mutation V111D. The membrane was depolarized to +120 mV from a potential of −100 mV. **E**) G–V relationship for the Hv1NC_HVRP1_ chimera compared to the G–V of Hv1 wild type and the Hv1NC_CiVSP_ chimera. Error bars are S.E.M., n = 5. Normalized conductances were measured from tail currents recorded in inside-out patches from oocytes. Curved line for Hv1NC_HVRP1_ represents a Boltzmann fit with parameters: V_1/2_ = 40±3 mV, and slope = 12.8±1.1 mV. Curves for Hv1 and Hv1NC_CiVSP_ represent previously published G-Vs with: V_1/2_ 53±3 mV, slope 11.6±0.6 mV, and V_1/2_ 68±2 mV, slope: 15.0±0.2 mV, respectively [16]. **F**) Example of deactivation kinetics of Hv1NC_HVRP1_ (teal) compared to Hv1 (red) and Hv1NC_CiVSP_ (orange). Tail currents were measured in response to a voltage step from +140 mV to −80 mV and normalized to the maximal current.

To learn more about the relationship between Hv1 and HVRP1, we compared the functional properties of the two proteins expressed in *Xenopus* oocytes, and then examined chimeric proteins in which parts of HVRP1 were swapped with the corresponding parts of Hv1 (Fig. 4A).

We measured large voltage-dependent proton currents from oocytes expressing the human Hv1 protein using two-electrode voltage-clamp (TEVC) (Fig. 4B–C). Under the same conditions, we could not detect any current from human HVRP1 (Fig. 4B–C), confirming a previous report in which a similar construct was expressed in HEK293 and COS-7 cells [30]. To enhance the proton current, the intracellular pH of the oocytes was lowered to pH 6.1 with 55 mM sodium acetate at pH 6.3 in the extracellular solution, as previously described [21], [40]. However, this did not have any measureable effect on HVRP1-expressing cells.

The lack of current from HVRP1 was not due to mislocalization of the protein in the oocyte. We labeled oocytes expressing an HVRP1 mutant with a substituted cysteine close to the extracellular end of S4 (S196C) with the thiol-reactive environment-sensitive dye TMRA-MTS [21]. From these oocytes, we were able to measure fluorescence changes in response to membrane depolarization using voltage-clamp fluorometry (Fig. 4D, central panel, black trace), indicating that the protein was on the plasma membrane. The magnitude of the fluorescence changes was similar to that observed with Hv1 labeled at the extracellular end of S4 (Fig. 4D, left panel, black trace). Oocytes expressing HVRP1 or Hv1 lacking cysteine substitutions did not show fluorescent changes (Fig. 4D, gray traces). In labeled Hv1, membrane depolarization causes a fast increase in fluorescence followed by a slower but more pronounced decrease. In labeled HVRP1 only the fast increase in fluorescence is observed upon depolarization (Fig. 4D central panel), suggesting that the environment around the fluorophore changes in different ways in the two proteins as a result of voltage change. We also found that the fluorescence of labeled HVRP1 was significantly reduced in the presence of the membrane-impermeable collisional quencher iodide (50 mM in the bath solution) both at negative and positive voltages (Fig. S2) supporting the interpretation that the change in fluorescence is linked to a voltage-depended conformational change of the protein and not to a direct effect of the transmembrane electric fields on the fluorophore. A similar result was previously obtained with the tetramethylrhodamine fluorophore attached to the outer end of the S4 segment of the Shaker potassium channel [41].

The fact that HVRP1 does not conduct current under voltage-clamp conditions could be due to the lack of a functioning conduction pathway in the VSD or to the failure in properly activating the protein under the experimental conditions used. While membrane depolarization is sufficient to open the activation gate of the Hv1 channel, some other stimuli might be required to activate HVRP1. We explored the possibility that the N- or the C-terminus of HVRP1 may be responsible for preventing the opening of the VSD gate in the absence of proper stimulation (e.g., binding to other proteins missing in the reconstituted system, incorrect post-translational modifications, etc.). When we exchanged the N- and C-termini of Hv1 with those of HVRP1 (Hv1NC_HVRP1_ chimera), we obtained functional channels for which we measured the conductance versus voltage relationship (G–V). We found that the G–V was shifted to less positive potentials compared to Hv1 wild type (Fig. 4E), indicating that VSD activation is facilitated in Hv1NC_HVRP1_. On the other hand, we could not measure any voltage-dependent current from the chimera in which the N- and C- termini of HVRP1 were replaced by the corresponding parts of Hv1 (HVRP1NC_Hv1_) (Fig. 4B–C) or from an HVRP1 construct with deleted N- and C- termini (HVRP1ΔNC, Fig. 4C). The substitution of cytoplasmic domains of Hv1 with the corresponding parts of CiVSP (Hv1NC_CiVSP_ chimera) is known to produce a G–V shift to more positive potentials and to strongly accelerate VSD deactivation compared to Hv1 wild type [16]. We found that in Hv1NC_HVRP1_, deactivation was not as accelerated as in Hv1NC_CiVSP_ (Fig. 4F). The smaller perturbation of VSD gating produced by the N- and C-termini of HVRP1 compared to CiVSP could be due to the fact that HVRP1 contains a coiled-coil domain similar to Hv1 [42].

In Hv1, aspartate D112 (located in the middle of the first transmembrane segment) has been proposed to be the channel’s selectivity filter [30]. Its substitution to a valine (the residue at the homologous position in HVRP1) was found to strongly reduce conduction and selectivity for protons [30]. We wondered whether the inverse substitution (V to D) in HVRP1 could confer proton permeability to its VSD. We generated HVRP1 and HVRP1-NC_Hv1_ constructs containing the V111D mutation (identified as HVRP1^V/D^ and HVRP1^V/D^NC_Hv1_, respectively) expressed them in *Xenopus* oocytes, and tested their conduction properties in two-electrode voltage clamp. We found that the constructs did not produce detectable currents under conditions in which Hv1 WT and Hv1NC_HVRP1_ were highly conducting (Fig. 4C shows the example of HVRP1^V/D^NC_Hv1_). Voltage-clamp fluorometry measurements performed on the labeled V111D mutants indicated that the proteins were targeted to the plasma membrane, albeit less effectively than the constructs lacking the V111D mutation (Fig. 4D).

Taken together, these findings indicate that: 1) the N- and C-termini of HVRP1 are compatible with VSD proton conduction. 2) The VSD of HVRP1 lacks a functional proton permeation pathway, despite the sequence similarity to Hv1. 3) Structural differences between the VSDs of Hv1 and HVRP1 prevent proton conduction in HVRP1 even in the presence of the S1 aspartate that forms the selectivity filter in Hv1.

**Figure 5:**
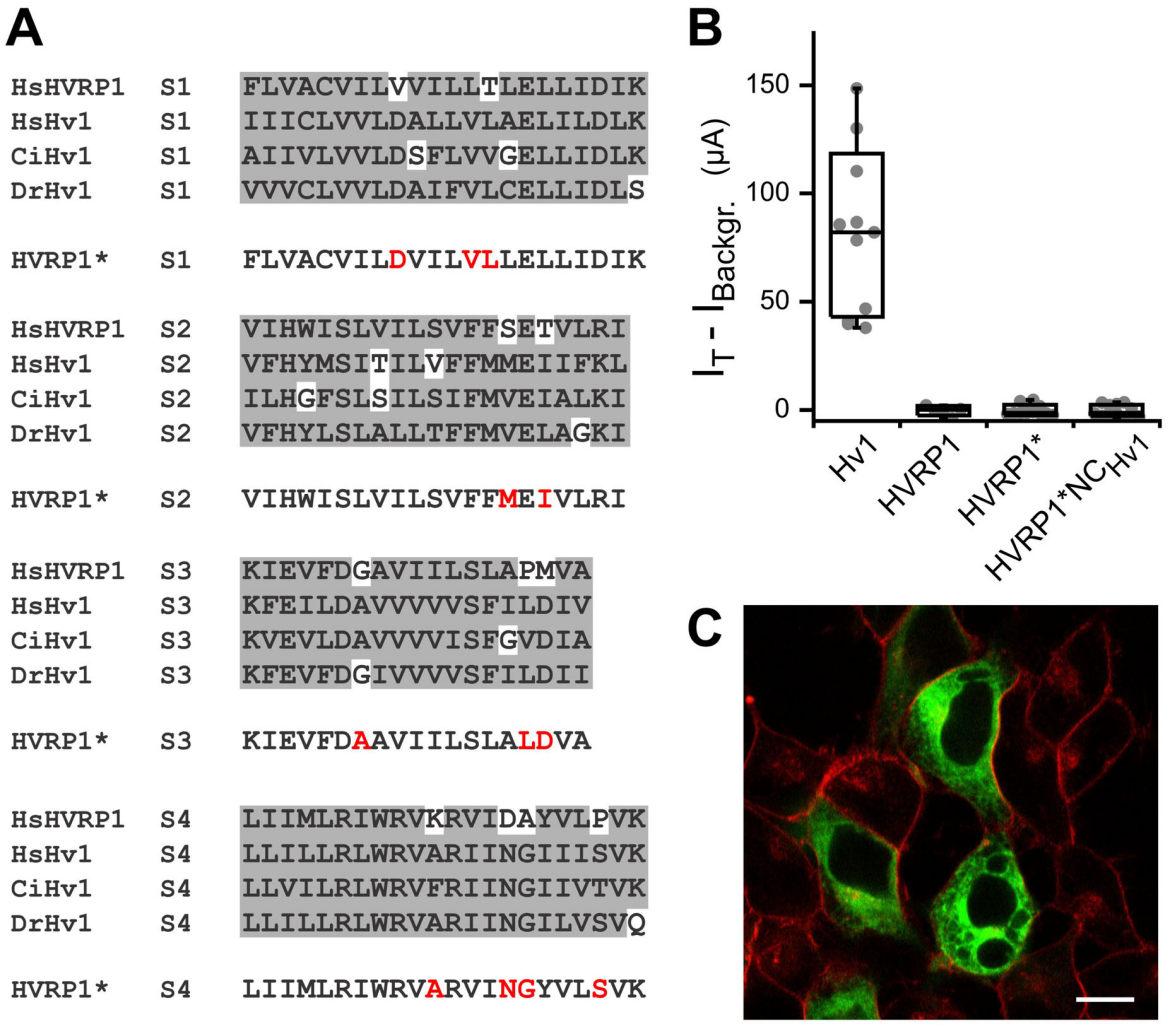
Consequences of increasing VSD similarity between Hv1 and HVRP1. **A**) Sequence alignments of transmembrane segments S1 through S4 of human HVRP1 and Hv1 channels from three different species. Residues of the same category (see text) are shown in gray background. Category mismatches have white backgrounds. Mutated residues in HVRP1* are shown in red. **B**) Quantification of test currents measured at +120 mV in oocytes expressing the indicated proteins (conditions as in Fig. 4B–C). Hv1 and HVRP1 are positive and negative controls, respectively. Box indicates median ± S.D., whisker shows range. Individual measurements are shown as gray circles, n = 5–12. **C**) Confocal image of live HEK293A cells expressing recombinant EGFP-tagged HVRP1* (green) and labeled with the plasma membrane marker FM-464 (red). Scale bar is 10 µm.

To further probe the similarity between the VSDs of Hv1 and HVRP1, we tested whether the functional properties of Hv1 could be transplanted into HVRP1 by altering residues in the HVRP1 transmembrane region that show poor conservation between HVRP1 and Hv1 but are highly conserved in Hv1 channels. To this end, we compared the sequences of the transmembrane segments of human HVRP1 with the corresponding segments of Hv1 channels from three distantly related species: *Homo sapiens*, *Danio rerio*, and *Ciona intestinalis* (Fig. 5A). We divided the amino acids in the S1–S4 segments into five categories: positively charged (R, K, H), negatively charged (D, E), polar non-charged (N, Q, S, T), hydrophobic (A, V, L, I, C, M, F, Y, W) and structural (G, P). We looked for category mismatches between residues of HVRP1 and the corresponding residues in the three Hv1 channels. Each mismatched residue in HVRP1 was then replaced with the corresponding residue of the human Hv1 by mutagenesis. We introduced a total of twelve mutations and refer to the mutated construct as HVRP1* (Fig. 5). Ten mutations (V111D, T116L, S151M, T153I, P184L, M185D, K213A, D217N, A218G, and P222S) corrected mismatched residues, and two additional mutations (L115V and G175A) further increased the similarity between HVRP1* and Hv1. HVRP1 L115 was replaced with valine because the homologous residue in human Hv1 (V116) was previously proposed to participate in proton conduction [43]. G175 was replaced with alanine to neutralize a potential helix break in close proximity to the highly conserved aspartate D174. We expressed HVRP1* in *Xenopus* oocytes and measured currents elicited by depolarization in TEVC. We could not detect any current higher than background (Fig. 5B). Replacing the N- and C- termini of HVRP1* with those of Hv1 did not change the result (Fig. 5B, HVRP1*NC_Hv1_). Fluorescently labeled oocytes expressing HVRP1* S196C did not produce measurable fluorescence changes suggesting that the construct might not be properly targeted to the plasma membrane. We expressed an EGFP-tagged version of HVRP1* in HEK293 and observed its subcellular distribution under confocal microscopy as we did for HVRP1 wild type. As shown in Fig. 5C, the subcellular compartmentalization of HVRP1* is different from that of HVRP1 wild type (Fig. 3G). The strong perturbation in protein trafficking caused by the twelve Hv1 residues transplanted into HVRP1 suggests that the VSD of this protein does not support the network of amino acid interactions present in Hv1, providing further evidence of the diversity between Hv1 and HVRP1.

## Discussion

The nervous system relies on proteins that can detect changes in membrane potential to generate and modulate electrical signals [44]. Accordingly, most of these proteins are highly expressed in the brain [45], and mutations in their genes produce neurological disorders [2], [46], [47]. All the known VSD-containing proteins involved in neuronal signaling are ion channels containing a pore domain. The voltage sensitive phosphatases and voltage-gated proton channels cloned so far are expressed primarily in non-excitable cells, and do not appear to perform functions specific to the nervous system [10], [11], [12], [13], [25], [26], [28].

Here we have identified HVRP1, a VSD-containing protein lacking a pore domain primarily expressed in the central nervous system. From a human gene expression profiling and qRT-PCR screening, HVRP1 was found to be highly expressed in cerebellar tissues. *In situ* hybridization on mouse brain revealed the presence of HVRP1 transcripts in the granule layer of the cerebellar cortex. Expression of the HVRP1 protein in cerebellar granule neurons in brain slices and cell culture was confirmed by IHC/ICC. Protein subcellular localization was then determined by immunogold electron microscopy. HVRP1 was detected at the dendritic claws of the granule cells surrounding glutamatergic mossy fiber terminals. We also found low levels of HVRP1 transcripts in few tissues outside the central nervous system, in particular in skeletal muscle and thyroid (Fig. 2B). Further studies are required to confirm expression at the protein level and to determine whether HVRP1 is uniformly distributed in these tissues or if it is enriched on the postsynaptic side of the end plate in muscles, or in cells contacting nerve terminals in the thyroid gland.Since the protein most closely related to HVRP1 is Hv1, we tested whether the two proteins had similar channel activity. We found that, unlike Hv1, HVRP1 did not conduct ions when expressed in *Xenopus* oocytes. We then tested chimeras between HVRP1 and Hv1 to establish whether the different behavior of the two proteins was due to the transmembrane VSD or to the N- and C-terminal cytoplasmic domains. We found that while the chimera Hv1-NC_HVRP1_ operated as an ion channel, the inverse chimera HVRP1-NC_Hv1_ did not. The HVRP1 construct lacking the N- and C- termini (HVRP1ΔNC) also failed to produce ionic currents. These findings indicated that, even though the VSD is the part of HVRP1 with the highest homology to Hv1, it is also the part responsible for the difference in ion channel activity between the two proteins. On the other hand, the N- and C-terminal domains of HVRP1 appear to be compatible with VSD gating and ion permeation.

We then found that the inability of HVRP1 to conduct ions did not depend on the presence of an aspartate residue in the middle of helix S1, whereas the corresponding residue in Hv1 was previously shown to act as the channel’s selectivity filter [30]. This suggested that the functional divergence between the VSDs of Hv1 and HVRP1 is not simply the result of a localized occlusion of the permeation pathway in HVRP1, but rather of a broader structural difference between the two domains. To further explore this structural difference, we created a hybrid HVRP1-Hv1 protein (HVRP1*) in which the four transmembrane segments contained the same set of polar, and positively and negatively charged residues found in Hv1 (Fig. 5A) and examined its ion conduction properties and cellular localization (Fig. 5B–C). Our results indicated that the set of Hv1 residues transplanted in HVRP1 were not well tolerated by the new background, a finding consistent with the idea that the structures of the VSDs of Hv1 and HVRP1 have diverged over time to perform distinct functions. Cerebellar granule neurons have rather short dendrites ending in glomerular structures that receive both excitatory inputs from glutamatergic mossy fibers and inhibitory inputs from GABAergic Golgi cells (Fig. 3C). The presence of HVRP1 on the dendritic plasma membrane of granule cells, and in particular in compartments rich in glutamate receptors and their regulatory proteins, suggests a function for HVRP1 related to the modulation of postsynaptic potentials and/or back-propagating action potentials. The presence of a VSD and a coiled-coil domain in HVRP1 and the lack of ion channel activity raise the possibility that the protein’s function is to provide voltage sensitivity to a distinct effector. The limited homology to known proteins of the HVRP1’s cytoplasmic region attached to the coiled-coil domain suggests that HVRP1 could affect neuronal physiology in a way not previously described in other VSD-containing proteins.

## Methods

All animal procedures for this study were reviewed and approved by the Institutional Animal Care and Use Committee (IACUC) at the University of California, Irvine. These procedures are consistent with the guidelines on euthanasia of the American Veterinary Medical Association (AVMA).

### DNA constructs for HVRP1 and Hv1-HVRP1 chimeras

Complementary DNA (cDNA) for human HVRP1/*C15ORF27* was custom synthesized by Epoch Biolabs. Mouse HVRP1/*AI118078* was cloned from mouse cerebellum cDNA (Zyagen). HVRP1 cDNAs were inserted in pGEMHE and pNICE vectors [48], [49], with or without C-terminal mEGFP tag. In the resulting fluorescent constructs, HVRP1 and mEGFP are connected by the flexible linker: SRGTSGGSGGSRGSGGSGG. Single point mutations, including the 12 mutations of HVRP1*, were introduced with standard PCR techniques. Chimeras between human Hv1 (IMAGE clone 5577070, Open Biosystems) and human HVRP1 were generated using the SOEing method [50]. Constructs were verified by full sequencing. In the Hv1NC_HVRP1_ chimera, residues 97–233 of HVRP1 were replaced by residues 98–230 of Hv1. In the HVRP1NC_Hv1_ chimera, residues 103–218 of Hv1 were replaced by residues 102–221 of HVRP1. The generation of chimera Hv1NC_CiVSP_ was previously described [16]. The sequence of the HVRP1ΔNC construct started with *M*KRAAVW and ended with VLPVKLE.

### Expression of recombinant HVRP1

For expression in oocytes, plasmids were linearized with NheI or SphI restriction enzymes (New England Biolabs) before *in vitro* transcription. RNA synthesis was carried out with a T7 mMessage mMachine transcription kit (Ambion). The correct sizes of the transcripts were confirmed by gel electrophoresis. mRNAs were injected in *Xenopus* oocytes (Ecocyte Bioscience) 1–3 days before the electrophysiological measurements (50 nl per cell, 0.3–1.5 µg/µl). Cells were maintained at 18°C in medium (ND96) containing 96 mM NaCl, 2 mM KCl, 1.8 mM CaCl_2_, 1 mM MgCl_2_, 10 mM HEPES, 5 mM pyruvate, 100 mg/L gentamicin, pH 7.2. HEK293A cells were cultured in DMEM (Life Technologies), supplemented with 10% fetal bovine serum (Gemini Bio-Products) and 1% Penstrep, at 37°C, under 5% CO_2_. Cells were transfected with pNICE constructs using Lipofectamine 2000 (Life Technologies).

### Culture of cerebellar granule neurons

Cerebellar granule cells were prepared from 7-day-old post-natal C57BL/6 mice (Charles River) as previously described [3]. Cells were cultured in Neurobasal A media supplemented with GlutaMAX (Life Technologies), penicillin, 250 µM KCl, and 2% B-27 (Life Technologies). To prevent the growth of non-neuronal cells, 10 µM of cytosine-β-D-arabinofuranoside was added 24 hours after the initial plating and with each subsequent media change. Cells were plated onto poly-D-lysine coated coverslips for immunohistochemistry.

### Microarray analysis

The body index of gene expression (BIGE) database was developed as previously described [38], [39]. This database was generated using total RNA from 4 male and 4 female human donors. The genome-wide gene expression data was obtained with Affymetrix Human Genome U133 Plus 2.0 gene arrays. Analysis of microarray data for genes *C15Orf27*/HVRP1, *HVCN1*, and *TPTE*, was performed with ArrayAssist software (Stratagene) and OriginPro 8.1 (OriginLab).

### Quantitative real-time PCR

Total RNA from human donors (Ambion & Clontech) was used to synthesize complementary DNA (cDNA) with a QuantiTect Reverse Transcription kit (Qiagen). The cDNA was then assayed with a Roche LightCycler 480 Real-Time PCR system, using the following HVRP1/*C15Orf27* primers: AGGGTGAAGAGGGTCATTGAT, and TCGTACTGCTGGATAACCATCTC. Data was collected using the LightCycler 480 software and analyzed by comparative C_T_ method using β-actin to normalize C_T_ values.

### Dendrogram

Genome databases were accessed at National Center for Biotechnology Information (NCBI) (http://www.ncbi.nlm.nih.gov/). The sequence of human HVRP1 was initially identified using the sequence of the voltage-sensing domain and C-terminus of human Hv1 as a search query on the BLASTP platform. To identify HVRP1 orthologs, the amino acid sequence of the human HVRP1 protein was used as a search query, using the default parameters of the BLASTP platform. Analysis of the resulting sequence data was performed with the Clustal program (Clustal Omega) (http://www.clustal.org/) on full-length proteins. Species abbreviations used can be found in Figure 1. The following proteins with NCBI and GenBank reference sequence numbers were used: ApHv1, XP_005028277.1; ApHVRP1, XP_005027974.1; ApTPTE2-like, XP_005014905.1; BtHv1, NP_001193182.1; BtHVRP1, XP_005222027.1; BtTPTE2-like, XP_005213782.1; CiHv1, NP_001071937.1; CiHVRP1, XP_002131775.1; CiVSP, NP_001028998.1; DrHv1, NP_001002346.1; DrHVRP1, NP_001074141.1; DrVSP, BAG50379.1; GgHv1, NP_001025834.1; GgHVRP1, XP_001233623.3; GgTPTE2, XP_417079.2; HsHv1, NP_001035196.1; HsHVRP1, NP_689548.2; HsTPTEα, NP_954870.2; HsTPTE2α, NP_570141.3; MbHv1, EPQ13961.1; MbHVRP1, XP_005874505.1; MbTPTE2-like, XP_005877021.1; MdHv1, XP_001372655.2; MdHVRP1, XP_001376486.2; MdPTEN-like, XP_001363283.1; MoHv1, XP_003738360.1; MoTPTE2-like, XP_003745380.1; OaHv1, XP_001505975.2; OaTPTE2-like, XP_001513133.2; OhHv1, ETE71598.1; OhHVRP1, ETE66651.1; OhTPTE2, ETE70810.1; OoHv1, XP_004276784.1; OoHVRP1, XP_004276399.1; OoTPTE2-like, XP_004274689.1; PsHv1, XP_006132641.1; PsHVRP1, XP_006126300.1; PsTPTE2-like, XP_006124968.1; SpHv1, NP_001119779.1; SpHVRP1, XP_003724918.1; SpTPTE2-like. XP_003731108.1; TtHv1, XP_004310881.1; TtHVRP1, XP_004319937.1; TtTPTE2-like, XP_004323024.1; XtHv1, NP_001011262.1; XtHVRP1, XP_004916038.1; XtPTEN2like, NP_001015951.1.

### *In situ* hybridization

Mouse HVRP1 cDNA fragments of 700 bp were inserted with opposite orientations in a pGEM vector between the T7 promoter and SphI restriction site using the SOEing technique and Phusion High-Fidelity polymeare (New England Biolabs). Sense and antisense digoxigenin-labeled RNA probes were prepared from SphI-linearized plasmids using the DIG RNA labeling mix from Roche. RNA probes were then cleaned and concentrated using a kit from Zymo Research. The cDNA sequence for the sense probe included the region starting with 5′-GAGCTTCTCATAGATA– and ending in –GAACCAGCAGTATGTG-3′. The corresponding reverse complement sequence was used as cDNA for the antisense probe. Adult C57BL/6 (Charles River) mice were deeply anesthetized with Nembutal and transcardially perfused with fixative (4% paraformaldehyde (PFA), 0.05% glutaraldehyde, and 0.2% picric acid dissolved in 0.1 M phosphate buffer, pH = 7.4) and saline. Brains were surgically removed following sacrifice, fixed overnight, washed with PBS and treated with 30% sucrose overnight. Then, they were embedded in OCT compound (Tissue-Tek) in plastic molds (Ted Pella) and frozen. Sagittal cryosections of 10–14 µm thickness were prepared using a Leica 3050S cryostat. Individual sections were collected and floated into sterile PBS during the cryosectioning process and mounted onto SuperFrost Plus slides (Fisher) using the free-floating method. Sections were positioned onto the slides with a fine paintbrush. They were allowed to air dry and adhere to the slides, and then used immediately. The procedure for the *in situ* hybridization was adapted from ref. [51]. The hybridization step and subsequent washing steps were performed in an Easy Dip Slide Staining System (Ted Pella). Samples were incubated with anti-digoxigenin antibody conjugated to alkaline phosphatase (Roche). The HVRP1 transcript was visualized by detection of digoxigenin by incubating samples overnight in a staining solution of Nitro blue tetrazolium (NBT) and 5-Bromo-4-chloro-3-indolyl phosphate (BCIP) solution (Roche). The staining reaction was stopped with 4% PFA. Sections were mounted with 70% glycerol under glass coverslips. Digital images were captured using DP Controller software (Olympus) connected to an upright Olympus SZX12 microscope.

### HVRP1 antibody

Two rabbits were immunized against the mouse HVRP1 C-terminal peptide EEKFRSLESKEPKLHTVPEA by Open Biosystems. Antisera were tested on Western blots against the recombinant HVRP1 expressed in oocytes and in immunohistochemistry of rat cerebellar tissue. The antiserum with the lowest background in both assays was purified by affinity chromatography (Open Biosystems) and used thereafter at dilutions 1∶500 or 1∶1000.

### Confocal imaging

HEK293A cells transfected with hHVRP1-EGFP or HVRP1*-EGFP in pNICE were grown on poly-D-lysine coated glass-bottom dishes (Mattek) for 1 to 2 days. Directly before imaging, live cells were washed in cold HBSS (Life Technologies) to remove residual FBS, and bathed in fresh HBSS. FM 4–64 dye (Life Technologies) was added to the solution (5 µM final concentration) to label the plasma membrane. Cells were imaged on a Zeiss LSM 780 confocal microscope with an LD C-Apochromat 63× immersion objective with 1.15 numeric aperture. For EGFP, excitation was at 488 nm, and emission band was 491–560 nm. For FM 4–64, excitation was at 561 nm, and emission band was 592–759. Primary cerebellar granule neurons growing on poly-D-lysine coated coverslips were fixated in 1∶1 methanol and acetone solution for ten minutes at −20°C. Immunocytochemistry was performed using either anti-HVRP1 antibody diluted 1∶1000, or anti-HVRP1 antibody diluted 1∶500 pre-incubated with peptide antigen (1∶500 dilution of a 1 µg/µl solution), and AlexaFluor-594-labeled goat anti-rabbit secondary antibody. Coverslips were then mounted onto Fisher SuperFrost Plus slides with ProLong Gold Antifade Reagent with DAPI (Life Technologies). For AlexaFluor 594, excitation was at 561 nm, and emission band was 592–759 nm. For DAPI, excitation was at 405 nm and emission band was 415–735 nm.

### Immunohistochemistry and electron microscopy

Adult Wistar rats were deeply anesthetized with ketamine-xilazine and transcardially perfused with ice cold saline for 1 minute then with fixative containing 4% paraformaldehyde 0.1% glutaraldehyde and 30% saturated picric acid dissolved in 0.1 M phosphate-buffer (PB). After overnight post fixation in the same fixative, brains were cut into 60 µm sections with a vibratome (Leica). Sections were treated with 0.1% sodium borohydrate in 0.1 M PB for 10 minutes, then extensively washed for 30 minutes in 0.1 M PB. Sections were thereafter incubated in 10% normal goat serum (NGS, Vector Labs), and then in 2% NGS and 1∶500 rabbit anti-hHVRP1 antibody for 48 hours at 4°C. After several washes in buffer, the sections were incubated in 0.8-nm-gold-conjugated goat anti-rabbit dissolved in 0.1 M PB containing 0.1% cold-water fish skin gelatin (Aurion) and 1% bovine serum albumin (Sigma-Aldrich) overnight at room temperature. The gold particles were increased in size with R-Gent silver enhancement kit (Aurion). The sections were thereafter treated with 1% OsO_4_ for 30 minutes, then contrasted with 1% uranil acetate, dehydrated and embedded in Durcupan (Sigma-Aldrich). Ultrathin sections (60 nm) were imaged with a Philips transmission electron microscope and pictures were taken with a Gatan camera.

### Electrophysiology and voltage-clamp fluorometry

Two-electrode voltage clamp and voltage-clamp fluorometry measurements were performed on *Xenopus* oocytes 1 – 3 days post-injection as previously described [21]. Bath solution contained either 100 mM NaCl, 3 mM CaCl_2_, 2.5 mM KCl, 5mM HEPES, pH = 7.4, or 55 mM sodium methanesulfonate, 55 mM sodium acetate, 10 mM MES, 2 mM MgCl_2_, pH = 6.3. For VCF recordings, native cysteines were blocked by incubating oocytes in glycine-maleimide (Toronto Research Chemicals) dissolved in ND96 for 30 minutes at room temperature 1–2 hours post-injection. Oocytes were labeled with 2-((5(6)-tetramethyl-rhodamine)carboxylamino)ethyl methanethiosulfonate (TAMRA-MTS) (Toronto Research Chemicals) in the dark for 1 minute at room temperature on the day of recording, and all labeled oocytes were stored at 10°C until use. An Oocyte Clamp OC-725C (Warner Instruments) was used to measure currents and control the membrane potential. A Dagan PhotoMax 200 coupled to a PIN photodiode was used to measure fluorescence signals. A CoolLED pE-2 excitation system (550 nm) connected to an Olympus IX71 microscope was used to excite the fluorophore. The oocyte membrane was visualized through a 20X Olympus UPlansApo objective (N.A. 0.75) and a Chroma TRITC filter cube. The amplifiers for current and fluorescence signals were interfaced to a PC through a 1440A Digidata analog/digital converter (Molecular Devices). Traces were filtered at 1 KHz and acquired at 5 KHz. Data were then analyzed with Clampfit 10.2 (Molecular Devices) and Origin 9 software (OriginLab). The effect of collisional quencher iodide on the fluorescence signal of TAMRA-MTS labeled HVRP1 S196C was measured by recording fluorescence changes as a result of membrane depolarization in the presence of 50 mM NaCl, 50 mM KCl, 3 mM CaCl_2_, 5mM HEPES, pH = 7.4 and then in the presence of a solution in which 50 mM KI replaced the 50 mM KCl. The negative changes in fluorescence intensity reported in Fig. S2 were corrected for photobleaching. Patch-clamp recordings were performed on excised inside-out patches from oocytes using an Axopatch 200B amplifier (Molecular Devices) as described in [52]. Both bath and pipette solutions contained 100 mM 2-(N-morpholino)ethanesulphonic acid (MES), 30 mM tetraethylammonium (TEA) methanesulfonate, 5 mM TEA chloride, 5 mM ethyleneglycol-bis(2-aminoethyl)-N,N,N′,N′-tetra-acetic acid (EGTA), adjusted to pH 6.0 with TEA hydroxide. Measurements were performed at 22±2°C with pipettes of 2–4 MΩ access resistance. Traces were filtered at 1 KHz and acquired at 5 KHz. They were then analyzed with Clampfit 10.2 (Molecular Devices) and Origin 9 (OriginLab).

### Western blotting

Total protein from transfected and non-transfected HEK293A cells was collected with lysis buffer (50 mM Tris-HCl, pH 7.5, 5 mM EDTA, 150 mM NaCl, 1% Triton-X 100, and protease inhibitor cocktail [Sigma]). The cell lysates were centrifuged at 13,000 rpm for 10 minutes at 4°C. Proteins from supernatants were separated by SDS-PAGE (4–20%, Tris-Glycine, Life Technologies). After gel electrophoresis, proteins were transferred to polyvinylidene difluoride (PVDF) membranes (Millipore). The membranes were incubated with anti-HVRP1 antibody at a 1∶1000 dilution, or anti-HVRP1 antibody neutralized by antigen peptide (1∶1000 dilution of a 1 µg/µl solution). Mouse anti-rabbit secondary antibody was conjugated with horseradish peroxidase (Chemicon International) and used at a 1∶50,000 dilution. The membranes were stripped by washing with a mild stripping buffer (1.5% glycine, 0.1% SDS, 0.5% Tween-20, pH 2.2) for 2×10 minutes, then PBS 2×10 minutes, and TBST 2×10 minutes. Membranes were then re-probed with anti-β-actin antibody conjugated to HRP (Abcam) at a 1∶5000 dilution. All proteins interacting with primary antibodies were visualized with Super Signal West Pico chemiluminescent substrate (Thermo Fisher).

## Supporting Information

Figure S1 **Predicted coiled-coil domain in HVRP1. A**) Human HVRP1 and Hv1 sequences were analyzed with the program Coils [Lupas, A., Van Dyke, M., and Stock, J. (1991) *Science* 252∶1162–1164] in ExPASy. The program predicts parallel two-stranded coiled-coil domains in the C-terminal regions right after the S4 transmembrane segments of the two proteins. The coiled-coil domain of Hv1 has been confirmed experimentally by X-ray crystallography [19], [20]. **B**) Homology model of the HVRP1 coiled-coil region crested using SWISS-MODEL [Arnold, K., Bordoli, L., Kopp, J., and Schwede, T. (2006) *Bioinformatics* 22∶195–201] in ExPASy. The structure of the Hv1 coiled-coil domain from ref. [20] (PDB code: 3 VMX) was used as a template. The alignment with the template is shown in (C). Representation of the structure was made in PyMOL (Schrödinger). **C**) Sequence of the coiled-coil domain of mouse Hv1 aligned with the predicted coiled-coil domain of HVRP1. Positions *abcdef* of the heptad repeat are shown above the alignment. Layer numbers and color scheme are as in ref. [20].
(TIF)

Figure S2 **Quenching effect of iodide ion on fluorescence from labeled HVRP1 S196C. A**) Fluorescence changes induced by depolarization measured from an oocyte expressing HVRP1 S196C labeled with TAMRA-MTS before (dark blue trace) and after (light blue) the addition of I^−^. Fluorescence is expressed as 100×(F(V)−F_−80 mV_)/F_−80 mV_. **B**) Quantification of the decrease in fluorescence produced by I^−^ at the indicated membrane potentials. Box indicates median ± S.D., whisker shows range. Individual measurements are shown as circles, n = 9. The variance of the fluorescence quenching reflects variability between labeled cells. In each individual cell, the quenching increased with membrane potential (p<10^−5^, paired-sample t-test, n = 9).
(TIF)

Table S1 **Tissue distribution of human HVRP1/C15ORF27, Hv1/HVCN1, and TPTE/PTEN2.** For each individual gene, absolute values were normalized to the maximum expression level (shown in blue) after median subtraction. Positive and negative values indicate up-regulated and down-regulated expression, respectively.
(DOCX)
