# Supplementary figures and images for: Evidence for Functional Diversity between the Voltage-Gated Proton Channel Hv1 and Its Closest Related Protein HVRP1
Source: PLoS One. 2014 Aug 28;9(8):e105926. doi: 10.1371/journal.pone.0105926 (PMC4148356; doi:10.1371/journal.pone.0105926)

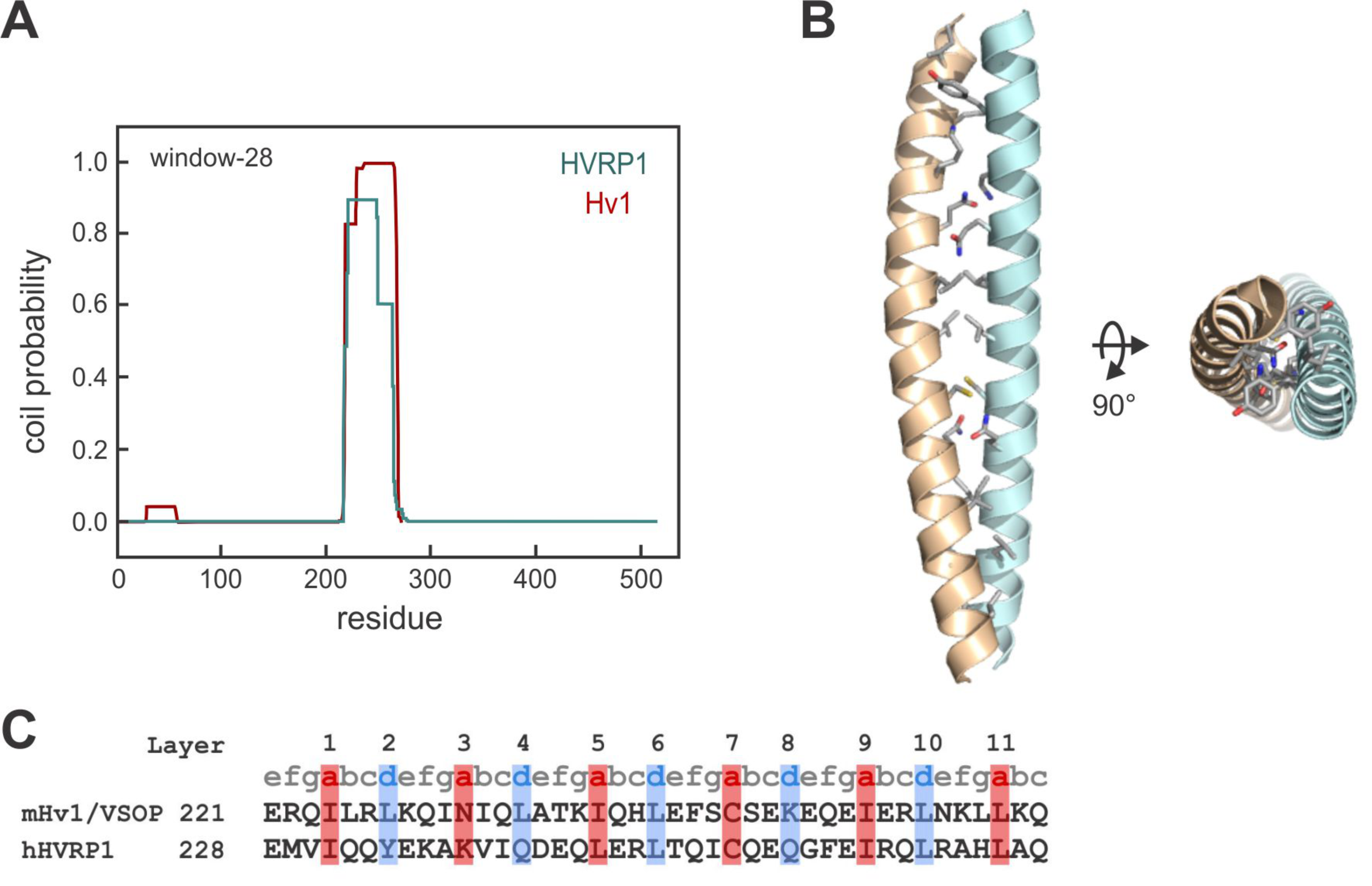

Supplement: Figure S1 — Predicted coiled-coil domain in HVRP1. A) Human HVRP1 and Hv1 sequences were analyzed with the program Coils [Lupas, A., Van Dyke, M., and Stock, J. (1991) Science 252∶1162–1164] in ExPASy. The program predicts parallel two-stranded coiled-coil domains in the C-terminal regions right after the S4 transmembrane segments of the two proteins. The coiled-coil domain of Hv1 has been confirmed experimentally by X-ray crystallography [19], [20]. B) Homology model of the HVRP1 coiled-coil region crested using SWISS-MODEL [Arnold, K., Bordoli, L., Kopp, J., and Schwede, T. (2006) Bioinformatics 22∶195–201] in ExPASy. The structure of the Hv1 coiled-coil domain from ref. [20] (PDB code: 3 VMX) was used as a template. The alignment with the template is shown in (C). Representation of the structure was made in PyMOL (Schrödinger). C) Sequence of the coiled-coil domain of mouse Hv1 aligned with the predicted coiled-coil domain of HVRP1. Positions abcdef of the heptad repeat are shown above the alignment. Layer numbers and color scheme are as in ref. [20]. (TIF) [file pone.0105926.s001.tif]

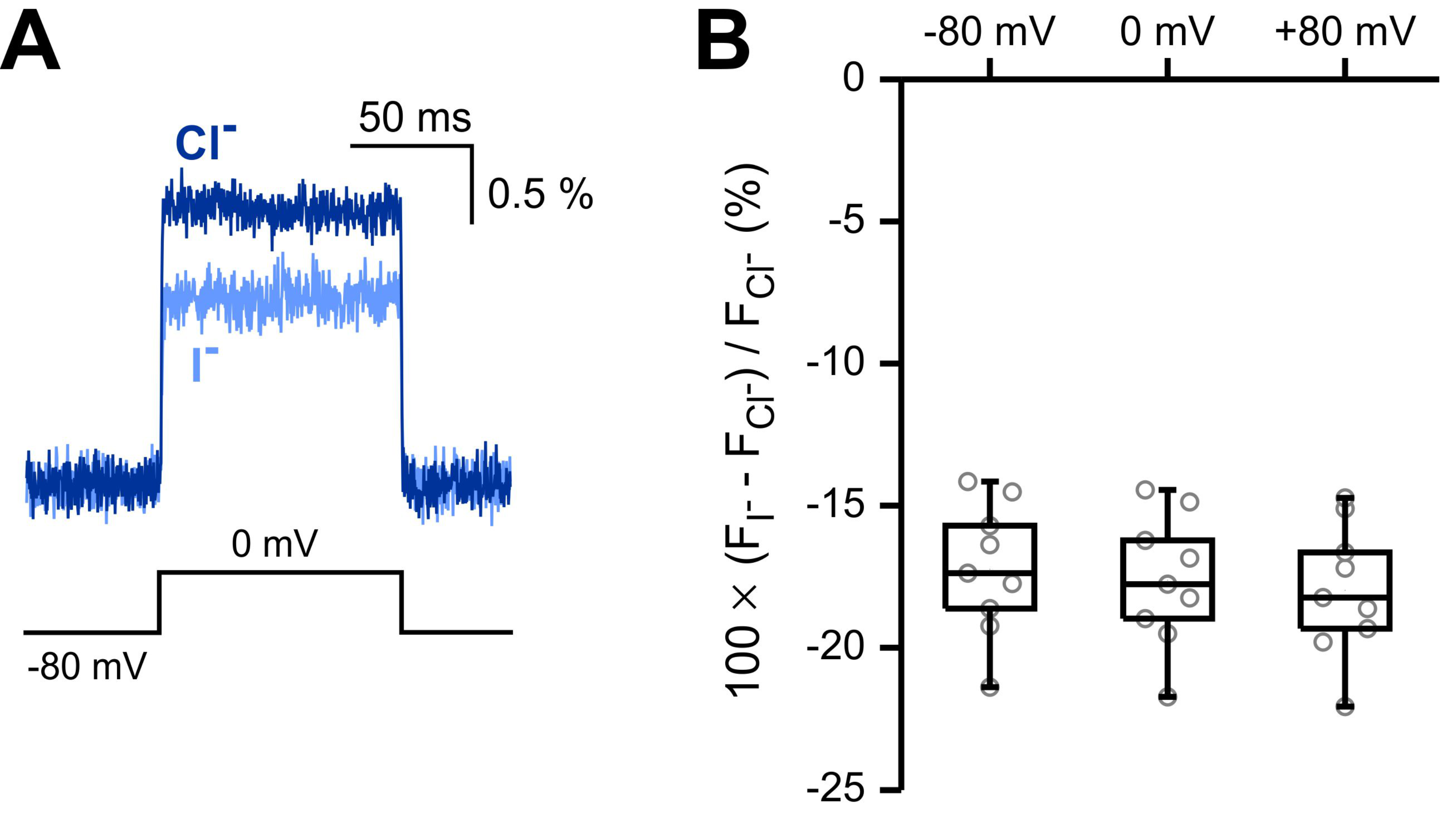

Supplement: Figure S2 — Quenching effect of iodide ion on fluorescence from labeled HVRP1 S196C. A) Fluorescence changes induced by depolarization measured from an oocyte expressing HVRP1 S196C labeled with TAMRA-MTS before (dark blue trace) and after (light blue) the addition of I−. Fluorescence is expressed as 100×(F(V)−F−80 mV)/F−80 mV. B) Quantification of the decrease in fluorescence produced by I− at the indicated membrane potentials. Box indicates median ± S.D., whisker shows range. Individual measurements are shown as circles, n = 9. The variance of the fluorescence quenching reflects variability between labeled cells. In each individual cell, the quenching increased with membrane potential (p<10−5, paired-sample t-test, n = 9). (TIF) [file pone.0105926.s002.tif]
