# Supplementary material for: Evidence for Functional Diversity between the Voltage-Gated Proton Channel Hv1 and Its Closest Related Protein HVRP1
Source: PLoS One. 2014 Aug 28;9(8):e105926. doi: 10.1371/journal.pone.0105926 (PMC4148356; doi:10.1371/journal.pone.0105926)
Supplement: Table S1 — Tissue distribution of human HVRP1/C15ORF27, Hv1/HVCN1, and TPTE/PTEN2. For each individual gene, absolute values were normalized to the maximum expression level (shown in blue) after median subtraction. Positive and negative values indicate up-regulated and down-regulated expression, respectively. (DOCX) [file pone.0105926.s003.docx]

**Table S1.**

| n | **Tissue** | **HVRP1/**  **C15ORF27** | **HVCN1/**  **MGC15619** | **TPTE/**  **PTEN2** |
| --- | --- | --- | --- | --- |
| 1 | Cerebral cortex | 0.1028 | -0.0382 | -5.12463E-4 |
| 2 | Prefrontal cortex | 0.00953 | -0.06775 | -4.03111E-4 |
| 3 | Frontal cortex | -0.05867 | -0.0395 | -5.8629E-4 |
| 4 | Frontal lobe | 0.05989 | -0.02112 | -1.45221E-4 |
| 5 | Parietal lobe | 0.06119 | -0.02778 | 1.33576E-4 |
| 6 | Occipital lobe | 0.06071 | -0.02341 | -4.90764E-4 |
| 7 | Temporal lobe | 0.06626 | -0.02386 | 9.30587E-4 |
| 8 | Corpus callosum | 0.02011 | 0.04222 | -1.61723E-4 |
| 9 | Accumbens | 7.1291E-4 | -0.02121 | -3.29741E-4 |
| 10 | Putamen | -0.00473 | -0.02332 | 7.3381E-10 |
| 11 | Globus pallidus | -0.12441 | 0.07486 | 1.74259E-4 |
| 12 | Amygdala | 0.09238 | -0.0361 | 2.56122E-5 |
| 13 | Hippocampus | 0.12182 | -0.01772 | -6.43837E-5 |
| 14 | Ventral tegmental area | 0.05941 | 0.0941 | 7.51509E-4 |
| 15 | Thalamus | 0.03427 | 0.04341 | 9.70315E-4 |
| 16 | Subthalamic nuclei | -0.00603 | 0.10989 | 6.16615E-4 |
| 17 | Hypothalamus | 0.0701 | 0.0744 | 1.71761E-4 |
| 18 | Cerebellar hemisphere | 0.35123 | -0.05444 | 0.00131 |
| 19 | Cerebellar vermis | 0.64944 | -0.01868 | 6.09891E-4 |
| 20 | **Cerebellum** | **1** | -0.04708 | 4.6069E-4 |
| 21 | Substantia nigra | 0.06297 | 0.12751 | 0.00119 |
| 22 | Midbrain | -0.00517 | 0.05388 | 0.00145 |
| 23 | Pons | 0.04834 | -0.0443 | -7.71975E-4 |
| 24 | Medulla | 0.00348 | 0.03542 | -1.54578E-4 |
| 25 | Vestibular nuclei superior | 0.01314 | 0.0348 | 9.15503E-4 |
| 26 | Nodose nucleus | 0.01364 | 0.07923 | 4.30438E-5 |
| 27 | Spinal cord | -0.01218 | 0.06345 | -2.85842E-4 |
| 28 | Dorsal root ganglia | -0.07158 | -0.0248 | 1.70288E-4 |
| 29 | Trigeminal ganglia | -0.07643 | -0.01194 | -2.51115E-4 |
| 30 | Oral mucosa | -0.06583 | -0.04181 | -0.00218 |
| 31 | Pharyngeal mucosa | -0.04185 | -0.0374 | 9.45631E-5 |
| 32 | Tongue | 0.11861 | 0.0034 | -3.15883E-4 |
| 33 | Tongue main corpus | 0.01689 | -0.03283 | 5.03468E-4 |
| 34 | Tongue superior | 0.01941 | 0.16346 | -0.00123 |
| 35 | Salivary gland | 0.0181 | -0.01487 | 4.24108E-4 |
| 36 | Esophagus | -0.01711 | -0.03642 | -0.00121 |
| 37 | Stomach | -0.05831 | -0.00742 | 6.63115E-5 |
| 38 | Stomach cardiac | 0.06758 | 0.00902 | 7.95397E-4 |
| 39 | Stomach fundus | -0.02056 | -0.00491 | -7.74737E-4 |
| 40 | Stomach pyloric | -5.49618E-8 | 0 | -2.74972E-4 |
| 41 | Small intestine duodenum | -0.04671 | 0.03013 | 7.54349E-5 |
| 42 | Small intestine jejunum | 0.23393 | 0.02189 | -1.5209E-4 |
| 43 | Small intestine | -0.02717 | -0.00448 | 5.867E-4 |
| 44 | Colon cecum | -0.02503 | 0.02288 | 3.20271E-4 |
| 45 | Colon | -0.03862 | -0.03335 | 1.13957E-4 |
| 46 | Liver | -0.02567 | -0.03361 | -6.39347E-5 |
| 47 | Adipose | -0.06076 | 0.02187 | -6.4118E-4 |
| 48 | Adipose omental | -0.05082 | 0.02954 | -2.94722E-4 |
| 49 | Adipose subcutaneous | -0.05001 | 0.09632 | 9.1209E-4 |
| 50 | Joint tissue synovium | -0.06515 | 0.01103 | -0.00108 |
| 51 | Skeletal muscle | 0.28103 | -0.00276 | 5.67782E-4 |
| 52 | Skeletal muscle-sup. Quad. | 0.14975 | 0.00361 | 0.00454 |
| 53 | Skin | 0.02511 | -0.04216 | -2.08378E-4 |
| 54 | Pericardium | -0.15885 | -0.05233 | 9.12675E-4 |
| 55 | Heart | 0.06282 | 0.01091 | -3.32965E-4 |
| 56 | Heart atrium | 0.00511 | 0.03273 | 4.77277E-5 |
| 57 | Heart ventricle | 0.12466 | 0.02073 | 7.67343E-4 |
| 58 | Aorta | -0.07828 | -0.00701 | -9.50775E-4 |
| 59 | Coronary artery | -0.08049 | 0.02606 | 8.78745E-5 |
| 60 | Saphenous vein | -0.06853 | -0.01143 | 5.36649E-4 |
| 61 | Vena cava | 0.04146 | 0.02372 | 0.00201 |
| 62 | Trachea | -0.00679 | -0.04172 | -7.31785E-4 |
| 63 | Bronchus | -0.07813 | 0.0065 | -7.85764E-4 |
| 64 | Lung | -0.08811 | 0.06728 | -7.65704E-4 |
| 65 | Adrenal gland cortex | 0.06495 | 0.00135 | -8.89138E-4 |
| 66 | Pancreas | 0.08974 | 0.00422 | 6.15043E-4 |
| 67 | Pituitary gland | 0.23849 | -0.01573 | 7.19812E-4 |
| 68 | Thyroid gland | -0.07697 | -0.02272 | -3.97022E-4 |
| 69 | Ovary | -0.1021 | 0.00343 | -0.00117 |
| 70 | **Testes** | 0.08721 | 0.39569 | **1** |
| 71 | Kidney | 0.15946 | -0.0311 | -0.00139 |
| 72 | Kidney cortex | 0.06141 | -0.07694 | -4.69458E-4 |
| 73 | Kidney medulla | 0.08781 | -0.03885 | -4.11375E-4 |
| 74 | Urethra | -0.12793 | -0.00957 | -8.66727E-4 |
| 75 | Fallopian tube | -0.13675 | -0.02268 | -0.0016 |
| 76 | Uterus | -0.04897 | 0.06935 | -3.76565E-4 |
| 77 | Myometrium | -0.09354 | -7.6435E-4 | -3.16721E-4 |
| 78 | Endometrium | -0.08684 | -0.00491 | -0.00137 |
| 79 | Cervix | -0.10549 | 0.00367 | -0.00104 |
| 80 | Vagina | -0.10939 | -0.01428 | -6.32884E-4 |
| 81 | Vulva | 0.01544 | 0.00984 | 2.42907E-4 |
| 82 | Mammary gland | -0.0841 | -5.80533E-4 | -1.44052E-4 |
| 83 | Nipple cross section | -0.03856 | -0.00921 | -0.00131 |
| 84 | Penis | -0.05551 | -0.00825 | -0.00125 |
| 85 | Prostate gland | -0.09386 | -0.0401 | -1.44072E-4 |
| 86 | Bone marrow | 0.00532 | 0.16588 | 5.15395E-4 |
| 87 | Thymus gland | -0.09151 | 0.02143 | -0.00115 |
| 88 | Lymph node | 0.01921 | 0.44154 | -0.00108 |
| 89 | Spleen | 0.00904 | 0.47385 | -0.00116 |
| 90 | Tonsil | -0.03892 | 0.42621 | 5.91467E-4 |
| 91 | PBMC media BB | -0.12826 | 0.16665 | -3.48472E-4 |
| 92 | PBMC PMA+ionomycin | -0.19097 | -0.04773 | 0.00232 |
| 93 | Monocytes resting-30h | 0.06389 | 0.44558 | 0.00219 |
| 94 | Monocytes LPS+IFNg-30h | 0.04055 | 0.14308 | 0.00147 |
| 95 | **B cells resting-30h** | 0.1388 | **1** | 0.00361 |
| 96 | B cells antiCD40+IL4-30h | 0.02876 | 0.9774 | 0.00126 |
| 97 | T cells resting-30h | -0.02438 | 0.09947 | 0.00219 |
| 98 | T cells antiCD3-30h | 0.07684 | 0.1135 | 0.00257 |
| 99 | CD4+ | -0.14257 | 0.04733 | 3.24902E-4 |
| 100 | CD4+ antiCD3+antiCD28 | -0.0634 | -0.05988 | -9.55343E-4 |
| 101 | CD8+ | -0.10564 | -0.00224 | -0.00171 |
| 102 | CD8+ antiCD3+antiCD28 | -0.02323 | -0.04484 | 0.00136 |
| 103 | Fetal liver | 0.02353 | 0.11673 | 4.25718E-4 |
| 104 | Fetal brain | 0.16881 | 0.002 | 1.31635E-4 |
| 105 | Placenta | -0.04602 | -0.03684 | 0.01122 |
